# Five-Year Retrospective Radiographic Analysis of Peri-Implant Bone Stability: A Comparison Between Two Socket Preservation Protocols and Spontaneous Healing

**DOI:** 10.3390/dj14090550

**Published:** 2026-09-01

**Authors:** Esteban Colombo, Enrica Giammarinaro, Maria Menini, Paolo Pesce, Nicola De Angelis

**Affiliations:** 1 Unit of Prosthodontics and Implant Prosthodontics, Department of Surgical Sciences and Integrated Diagnostics, University of Genoa, 16126 Genova, Italy; 2 Department of Stomatology, Tuscan Dental Institute, 55041 Lido di Camaiore, Italy

**Keywords:** socket preservation, marginal bone loss (MBL), dental implants, barrier membrane, cortico-cancellous bone

## Abstract

Objectives: The primary objective of this retrospective pilot study was to evaluate the long-term radiographic stability of peri-implant marginal bone levels (MBLs) at a five-year follow-up, comparing two different socket preservation protocols against spontaneous healing. Materials and Methods: A total of 24 patients who underwent tooth extraction followed by delayed implant placement were divided into three groups of 8 subjects each. Test Group 1 was treated with a heterologous cortico-cancellous bone graft covered by a slow-resorbing bovine pericardium membrane. Test Group 2 received the same bone graft but covered by a rapidly resorbing native porcine collagen membrane. The Control Group was left to heal spontaneously. Marginal bone loss (MBL) was calculated radiographically by measuring the difference in bone levels between the time of osseointegration (T0) and the 5-year follow-up (T1). For each implant, mesial and distal MBL values were averaged to obtain a single mean per-implant MBL value for group-level analysis. Results: The 5-year implant survival rate was 100% across all analyzed groups. Test Group 1 demonstrated the highest peri-implant bone stability, recording a mean MBL of 0.17±0.20 mm. In contrast, Test Group 2 and the Control Group exhibited greater resorption, with mean MBL values of 0.46±0.12 mm and 0.45±0.27 mm, respectively. Descriptive analysis indicated a trend toward improved peri-implant bone preservation in the group treated with biomaterial and a slow-resorbing pericardium membrane compared to both the short-resorbing collagen membrane group and the spontaneous healing control group. Conclusions: Within the limitations of this pilot study, the results suggest that the choice of barrier membrane in socket preservation procedures could affect 5-year marginal bone stability. The application of a slow-resorbing membrane provided a prolonged barrier action and showed a descriptive trend of lower mean marginal bone loss. However, the extremely small sample size precludes establishing clinical superiority over standard collagen membranes or spontaneous healing. Further randomized clinical trials with larger cohorts are necessary to confirm these long-term findings.

## 1. Introduction

Tooth extraction is a routine procedure in oral surgery, yet it triggers a cascade of inevitable biological events that lead to significant structural alterations of the alveolar ridge. It is well-established in the literature that the alveolar process is a tooth-dependent structure; consequently, the loss of a dental element inevitably results in atrophic changes. As demonstrated by the pivotal work of Araújo and Lindhe [1], this remodeling is largely driven by the resorption of the bundle bone—the portion of the alveolar bone into which the periodontal ligament fibers are anchored. Following extraction, the bundle bone loses its function and vascular supply, leading to its rapid resorption, which is particularly pronounced at the buccal aspect of the ridge.

Systematic reviews indicate that untreated extraction sockets may lose up to 50% of their width within the first 12 months, with the most dramatic reduction occurring in the first 3 months [2,3]. The clinical implications of this volumetric contraction vary significantly depending on the location of the extraction site. In the anterior aesthetic zone, even minor horizontal resorption can lead to the collapse of the soft tissue architecture, compromising the final aesthetic outcome and making it challenging to achieve a harmonious emergence profile for the future implant-supported restoration [4]. Conversely, in the posterior sectors, while the aesthetic demand may be lower, the horizontal and vertical bone loss can critically reduce the available bone volume, often necessitating complex reconstructive procedures such as sinus lifting or guided bone regeneration (GBR) to allow for proper implant placement.

To counteract these adverse dimensional changes and simplify future rehabilitation, socket preservation—also referred to in the literature as alveolar ridge preservation (ARP)—has been proposed as a predictable therapeutic modality. The primary objective of socket preservation is not necessarily to induce new bone formation beyond the skeletal envelope but to minimize the volumetric shrinkage of the ridge and preserve the existing hard and soft tissue architecture [5]. The standard protocol for this technique involves the insertion of a biomaterial into the fresh extraction socket to act as a scaffold, which is then covered by a barrier membrane to prevent epithelial down-growth and stabilize the blood clot.

Regarding the biomaterials, deproteinized bovine bone mineral (DBBM) is among the most extensively documented xenografts due to its osteoconductive properties and slow resorption rate, which allows it to maintain the mechanical space effectively over time [6]. This is almost invariably combined with a resorbable membrane, such as a native collagen membrane or a pericardium matrix, which seals the socket and protects the graft during the early phases of healing. Recent consensus reports and clinical studies confirm that while socket preservation cannot completely halt the physiological resorption process, it significantly limits the horizontal and vertical bone loss compared to spontaneous healing, thereby reducing the need for further augmentation at the time of implant placement [7,8]. Histological evidence supports the efficacy of these procedures. Different scientific papers [9,10,11] have analyzed cohorts of patients treated with socket preservation, demonstrating that the use of xenografts results in the formation of a composite network of new vital bone and residual graft particles. These findings confirm that the grafted sites are biologically capable of supporting implant placement. However, while the histological and early volumetric outcomes are well documented, there is an ongoing debate in the scientific community regarding the long-term behavior of these grafted sites under functional loading.

The ultimate success of implant therapy is defined not only by successful osseointegration but also by the long-term stability of the peri-implant tissues. A critical parameter for this stability is marginal bone loss (MBL). MBL is a key indicator of peri-implant health, influenced by factors such as surgical trauma, the establishment of biological width, and the nature of the recipient bone [12]. Some authors have raised concerns that residual non-resorbable graft particles might interfere with bone-to-implant contact (BIC) or act as a risk factor in the event of peri-implant mucositis [13]. On the other hand, other researchers argue that the “composite bone” achieved through socket preservation offers superior resistance to resorption due to the low turnover rate of xenograft particles, potentially maintaining the buccal bone wall more effectively than native bone alone [14,15].

Despite the abundance of literature on the immediate post-extraction phase, few studies have provided a retrospective radiographic analysis with a medium-to-long-term follow-up that directly compares different socket preservation materials against a control group of spontaneous healing. Most existing data focus on volumetric changes prior to loading or report only short-term survival rates. Understanding whether the initial benefits of preserving the socket translate into radiographically stable bone levels after many years of function is crucial for validating the long-term prognosis of these treatments.

Therefore, the aim of this retrospective pilot study is to analyze peri-implant bone stability in a cohort of patients five years after implant placement. Specifically, this study aimed to quantify marginal bone loss radiographically, comparing sites treated with two different socket preservation protocols using distinct membranes versus a control group with spontaneous healing. This analysis sought to determine whether the type of healing modality influenced the long-term maintenance of bone levels and to verify whether grafted sites exhibited different resorption patterns compared with non-grafted sites under functional loading over time.

## 2. Materials and Methods

### 2.1. Study Design and Setting

This study was designed as a retrospective, monocentric, observational cohort pilot study. The investigation was conducted at a private specialist clinic, analyzing data from patients treated between January 2019 and December 2020. The 5-year follow-up examinations were performed between March and May 2026. All surgical and prosthetic procedures, as well as follow-up appointments, were performed by two experienced surgeons belonging to the same clinical center to ensure consistency in the surgical technique and protocol application.

Given the exploratory nature of the study and the limited sample size available after applying strict inclusion criteria, the results should be interpreted as preliminary and hypothesis-generating. The study was conducted in accordance with the ethical principles of the Declaration of Helsinki. All patients signed a written informed consent form prior to the surgical phases. The study was approved by the CERA ethics committee for research at Genoa University, with protocol number 2026/40.

### 2.2. Study Population and Selection Criteria

The study population was recruited from a pool of patients who underwent tooth extraction followed by delayed implant placement. Inclusion criteria were age between 30 and 80 years, ability to understand and sign a written consent form, indication for simple extraction in the posterior zone, need for delayed implant restoration with a standardized two-stage approach, presence of optimal healed soft tissue conditions with an adequate band of keratinized mucosa prior to implant placement, documented compliance with follow-up visits, light smoking habit of fewer than 10 cigarettes/day, and absence of periodontal disease or inclusion in periodontal maintenance therapy.

Exclusion criteria were teeth requiring surgical extraction or presenting with acute infection or extensive bone defects, uncontrolled systemic diseases, heavy smoking habits of more than 10 cigarettes/day, history of bisphosphonate therapy or head and neck radiation, and lack of standardized radiographic follow-up. Regarding the patient allocation process, it must be noted that this is a retrospective analysis of previously treated patients. At the time of the original surgical interventions, treatment allocation was not formally randomized. However, the choice between the two socket preservation protocols or spontaneous healing was not dictated by the specific clinical characteristics or the severity of the extraction site. Instead, the selection was based on the operator’s preference and the availability of the biomaterials at the time of surgery. All included sites met the same baseline inclusion criteria for simple extractions in the posterior zone. Consequently, the allocation to a specific treatment group was not influenced by a priori anatomical disadvantages, thereby mitigating the potential risk of selection bias.

### 2.3. Surgical Protocol: Socket Preservation

**Figure 1:**
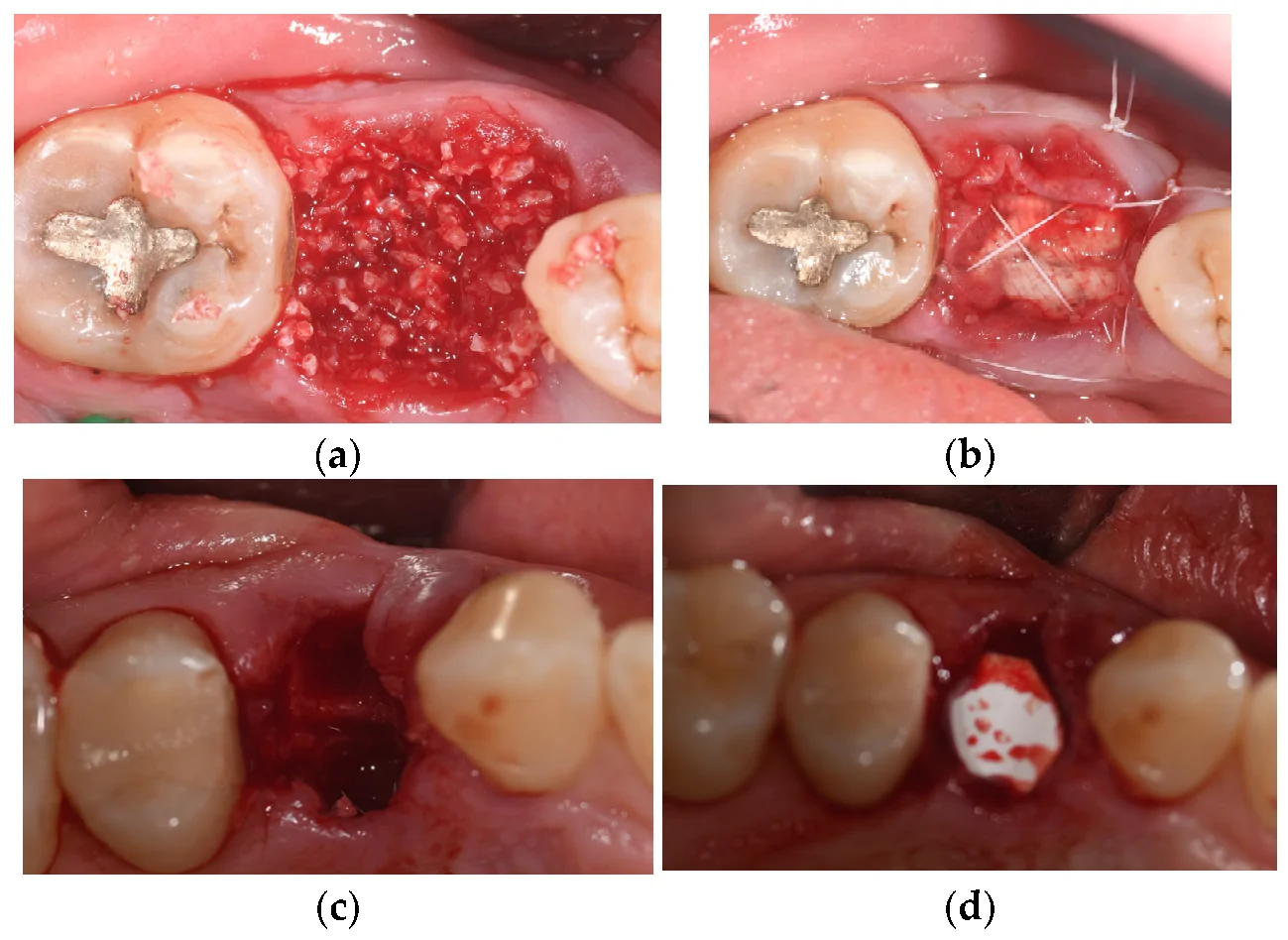
Clinical images of the surgical protocol for socket preservation: (**a**,**b**) In Test Group 2, a native porcine collagen membrane was used to seal the socket, while (**c**,**d**) in Test Group 1, the collagenic pericardium membrane was employed.

Patients included in the test groups underwent a standardized socket preservation procedure. Following atraumatic tooth extraction and careful debridement of the alveolus, the designated biomaterial was packed into the socket. Test Group 1 was treated with a cortico-cancellous heterologous bone graft covered by a slow-resorbing bovine pericardium membrane. Test Group 2 received the same graft covered by a native porcine collagen membrane. The Control Group underwent spontaneous healing without any grafting material or membrane. The surgical site was closed using 4/0 sutures (PGA or PTFE) to stabilize the biomaterial and the clot. Post-operative care included systemic antibiotic therapy and chemical plaque control using 0.20% chlorhexidine mouthwash until suture removal (Figure 1).

### 2.4. Implant Placement

Two implant systems were used in the present study: BTK IS+^®^ and Straumann BLT^®^. Both systems are tapered implants characterized by comparable macro-geometry, internal conical connection, and platform-switching configuration. Implant placement was performed according to the respective manufacturer’s surgical protocols. To minimize prosthetic-related confounding factors affecting marginal bone levels, all implants were restored using screw-retained prosthetic rehabilitations, and no cement-retained restorations were delivered.

### 2.5. Implant Stability Assessment

Implant stability following osseointegration was evaluated using the Osstell device (Osstell AB, Gothenburg, Sweden). The Implant Stability Quotient (ISQ) measurements were recorded at the end of the healing period to confirm successful osseointegration prior to prosthetic loading.

### 2.6. Radiographic Acquisition and Analysis

**Figure 2:**
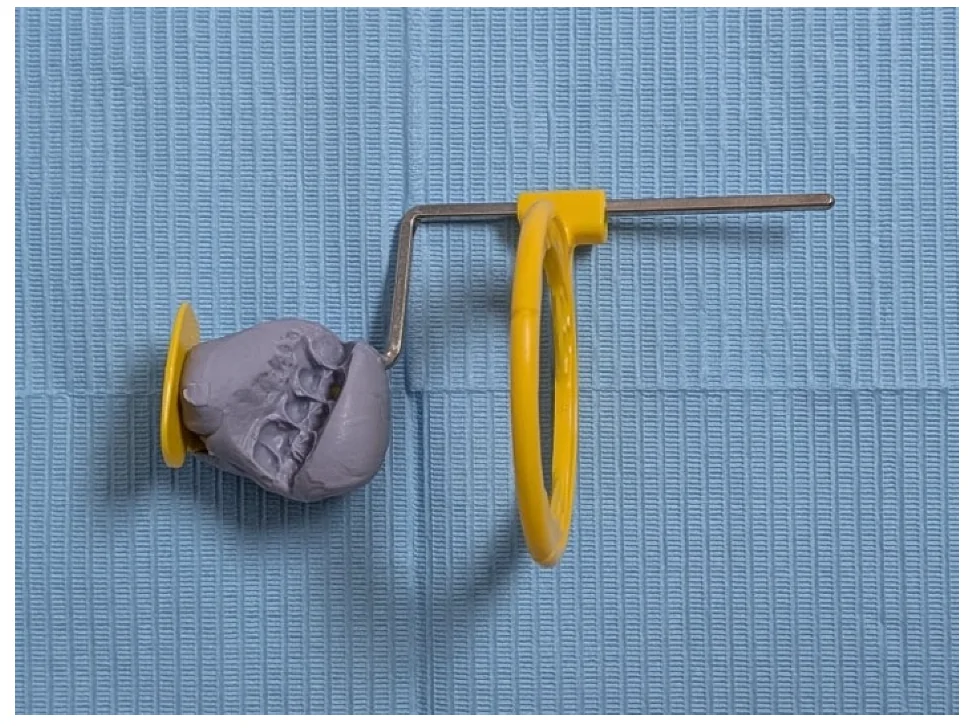
Example of the customized X-ray holder.

**Figure 3:**
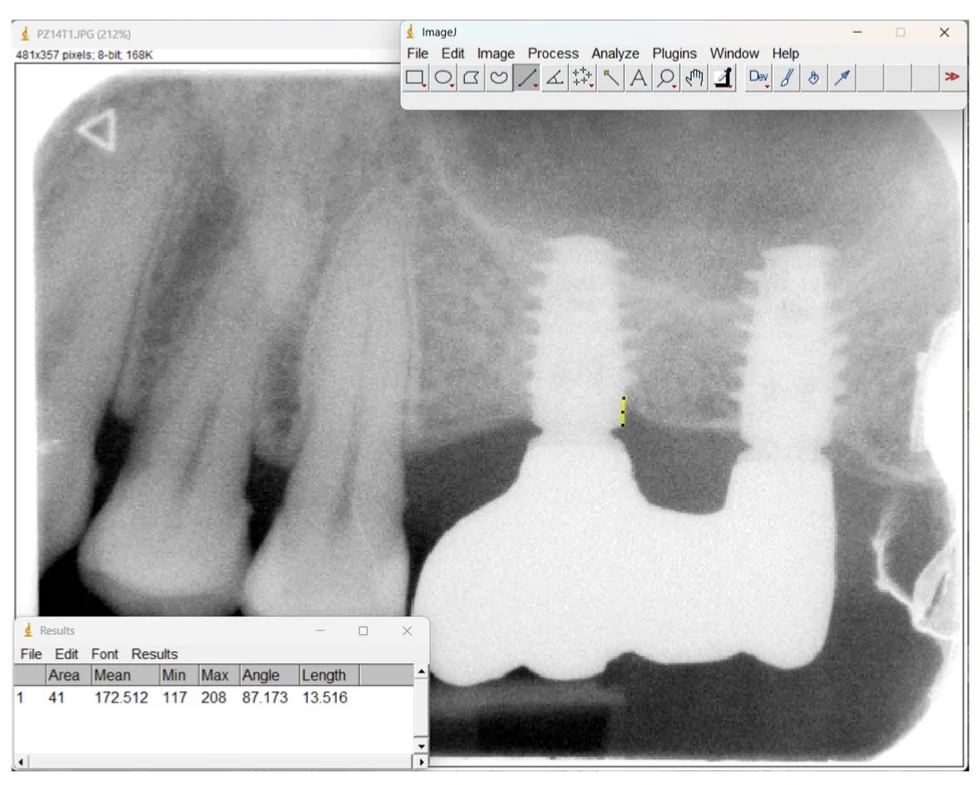
Example of radiographic analysis using ImageJ.

Radiographic evaluation was performed using digital intraoral periapical radiographs obtained with phosphor plate technology (PSP). To ensure reproducibility, images were acquired using a Rinn yellow holder customized with a silicone index (Figure 2), allowing identical projection geometry at different time points. Image analysis was conducted using ImageJ software vers 1.54r (National Institutes of Health, USA). Measurements were calibrated using the known technical specifications of the implant to convert pixels into millimeters (Figure 3).

All radiographic measurements were performed by a single trained examiner who was not involved in the surgical procedures. To assess intra-examiner reliability, measurements were repeated on a randomly selected subset of radiographs (20% of the sample) after a two-week interval. The intra-class correlation coefficient (ICC) for linear measurements was >0.90, indicating excellent intra-examiner agreement.

### 2.7. Assessment of Marginal Bone Loss

The primary outcome of the study was the evaluation of peri-implant bone stability. The most coronal point of the implant collar was selected as the fixed reference landmark (implant shoulder, IS). This point served as the zero reference point for calculating the linear distance to the first bone-to-implant contact (BIC). Measurements were taken at two time points: T0, defined as the radiograph taken after the healing period to confirm osseointegration and before prosthetic loading. It must be explicitly noted that T0 represents the baseline bone level established around the integrated implant and does not evaluate the initial crestal bone volume prior to tooth extraction. T1 was defined as the radiograph taken 5 years (±3 months) after implant placement. The MBL for each site (mesial and distal) was calculated as the difference in bone level between T1 and T0. For each implant, mesial and distal MBL values were averaged to obtain a single mean per-implant MBL value, which was used for group-level statistical analysis.

### 2.8. Power Analysis

Given the retrospective and exploratory nature of this pilot study, no prior sample size calculation was performed.

### 2.9. Statistical Analysis

Data collection was performed using a digital spreadsheet (Microsoft Excel). Due to the very small sample size (*n* = 8 per group), the study was strictly designed as an exploratory, hypothesis-generating investigation. Consequently, the statistical approach was restricted to a comprehensive descriptive analysis, as performing inferential statistical tests on such limited data would lack adequate statistical power and carry a high risk of misleading conclusions. Continuous variables, including patient age, implant insertion torque, and marginal bone loss (MBL), are expressed as mean ± standard deviation (SD) and 95% confidence intervals (CIs) to perform a descriptive statistical analysis of the clinical and radiographic outcomes.

## 3. Results

### 3.1. Study Population and Clinical Outcomes

A total of 24 patients (13 male and 11 female) were enrolled in the study and distributed into three groups of 8 subjects each (*n* = 24). The mean age of the study population was 52 ± 10.20 years, and approximately 30% of the patients were light smokers, as per the inclusion criteria. A total of 24 implants were placed in upper (*n* = 13) and lower (*n* = 11) jaws, specifically in premolar and molar sites, comprising 9 BTK IS+^®^ and 15 Straumann BLT^®^, as shown in Table 1.

The mean insertion torque was 26.9 ± 6.3 N. No implant failures occurred during the 5-year observation period, resulting in an overall cumulative survival rate of 100% across all groups. Given the limited sample size of this pilot investigation, this survival rate should be interpreted with caution. Regarding biological complications, the overall incidence was low, with a total of 4 cases of peri-implant mucositis. Specifically, these complications were observed in 2 patients in the Control Group (25%), 1 patient in Test Group 1 (12.5%), and 1 patient in Test Group 2 (12.5%). No advanced signs of peri-implantitis were detected in any site. All this data is summarized in Table 2.

Table 3 summarizes the clinical outcomes observed in the three study groups. All implants remained functional throughout the observation period, resulting in a survival rate of 100% across groups. Marginal bone loss (MBL) was evaluated radiographically by measuring mesial and distal bone level changes between baseline (T0) and follow-up (T1). Descriptive analysis revealed lower mean MBL values in Test Group 1 compared with Test Group 2 and the Control Group, while complication rates were low in all groups. These findings provide an overview of the radiographic peri-implant bone response associated with the different treatment protocols. Important clinical parameters of peri-implant health, such as probing depth, bleeding on probing (BOP), plaque accumulation, and soft tissue conditions, were not evaluated in this retrospective analysis.

### 3.2. Primary Outcome: Marginal Bone Loss (MBL)

The primary outcome of the present study was marginal bone loss (MBL) at the 5-year follow-up. The descriptive statistical analysis of the radiographic parameters is summarized in Table 4. Test Group 1 demonstrated the highest peri-implant bone stability among all groups, with a mean MBL of 0.17 ± 0.20 mm (95% CI: 0.00–0.34). In contrast, Test Group 2 and the Control Group exhibited greater bone resorption, with mean MBL values of 0.46 ± 0.12 mm (95% CI: 0.36–0.57) and 0.45 ± 0.27 mm (95% CI: 0.23–0.68), respectively. Although no formal inferential statistical analysis was performed because of the limited sample size, these descriptive findings suggest a trend toward improved peri-implant bone preservation in the group treated with biomaterial and pericardium membrane.

### 3.3. Secondary Outcome: Insertion Torque

Insertion torque was recorded at the time of implant placement as an indicator of primary implant stability. Descriptive statistics are summarized in Table 5. The highest mean insertion torque was observed in Test Group 1 (28.75 ± 6.94 Ncm), followed by Test Group 2 (26.88 ± 5.94 Ncm) and the Control Group (25.00 ± 6.55 Ncm). Although higher mean values were recorded in the grafted groups, the differences among groups were limited and should be interpreted cautiously given the small sample size. Overall, all groups achieved insertion torque values compatible with satisfactory primary implant stability.

## 4. Discussion

The primary objective of this retrospective pilot study was to evaluate the long-term radiographic stability of peri-implant marginal bone loss five years after implant placement, comparing two different socket preservation protocols that used the same cortico-cancellous bone graft but different barrier membranes against spontaneous healing. Within the limitations of this pilot study, a difference in the data was observed among the groups, suggesting that the type of barrier membrane may influence long-term maintenance of crestal bone. Specifically, sites treated with a slow-resorbing bovine pericardium membrane (Test Group 1) exhibited superior marginal stability (MBL = 0.17 mm) compared to both the native porcine collagen membrane group (Test Group 2, MBL = 0.46 mm) and the spontaneous healing control group (MBL = 0.45 mm). However, due to the extremely small sample size (8 implants per group), these observations remain purely descriptive and highly susceptible to random variation, strictly preventing any claims of statistical or clinical superiority. Additionally, the present study relies exclusively on radiographic marginal bone loss to assess outcomes. Because vital clinical indicators such as probing depth, BOP, and soft tissue profiles were not recorded, the conclusions of this investigation must be strictly restricted to radiographic bone stability rather than overall peri-implant health. While both test groups utilized the same heterologous cortico-cancellous bone graft, the discrepancy in MBL at 5 years highlights the relevance of the barrier function during the early healing stages. Native collagen membranes are known for their excellent biocompatibility but possess a relatively rapid degradation profile of approximately 4 to 6 weeks. In contrast, bovine pericardium membranes offer a prolonged barrier effect of up to 3 to 4 months. The premature degradation of the collagen membrane in Test Group 2 may have allowed early soft tissue invagination or crestal graft resorption before complete bone consolidation could occur. This concept is supported by recent literature investigating histological and dimensional changes of the alveolar ridge following extraction, emphasizing that hard-tissue stability is closely linked to barrier quality and soft-tissue sealing [16]. A longer-lasting barrier likely protects the slow-remodeling cortico-cancellous graft more effectively during the critical phase of woven bone formation, translating into greater crestal stability under functional loading years later.

The lack of differences in MBL between Test Group 2 and the Control Group at the 5-year mark warrants careful consideration. This finding is consistent with systematic reviews and long-term clinical trials that similarly observed no significant differences in marginal bone levels between preserved and non-preserved sockets in the long term [17]. This suggests that while standard socket preservation protocols with rapidly resorbing membranes may help maintain initial ridge dimensions, they may not always translate into a superior long-term radiographic advantage for crestal bone maintenance over an extended functional period compared with natural healing. When evaluating implant success, MBL is a primary indicator of peri-implant health. The excellent stability observed in Test Group 1 aligns with 5-year results reporting mean MBL values of approximately 0.18 ± 0.14 mm in preserved sites [18]. Looking at even longer follow-ups, 10-year outcomes of implants placed in preserved ridges demonstrate that, after the early period of remodeling, bone levels may remain stable over time [19]. It is important to note that, according to standard success criteria, all groups in the present study exhibited clinically acceptable MBL values at 5 years [12].

Despite the lack of a difference in MBL between Test Group 2 and the Control Group, the overall clinical rationale for socket preservation remains strong. Socket preservation predictably minimizes horizontal and vertical volumetric shrinkage of the alveolar ridge compared with extraction alone [20]. This concept has been corroborated by more recent investigations reporting favorable outcomes of alveolar ridge preservation approaches [21]. Regarding secondary outcomes, the present study observed a trend toward higher insertion torque in grafted sites compared with spontaneously healed sites. Experimental studies have reported that socket preservation may not significantly alter implant primary stability compared with natural healing [22], suggesting that the relationship between graft maturation, bone density, and mechanical stability remains complex.

The findings of the present investigation must be interpreted in light of its limitations. This study was designed as a pilot retrospective analysis with a very small sample size of 8 implants per group. This restricted cohort considerably limits the robustness and generalizability of the results, reinforcing the strictly exploratory and hypothesis-generating nature of this investigation. The considered decision to forgo inferential statistical analysis in favor of descriptive statistics reflects this fundamental limitation. Furthermore, although surgical and prosthetic procedures were standardized as much as possible, residual confounding related to patient-level and site-level factors cannot be excluded. A major limitation of this investigation is its retrospective design. Because the analysis was limited to the available patient population rather than a strictly controlled cohort typical of a randomized clinical trial (RCT), patient allocation was not randomized. Consequently, treatment selection may have been influenced by the operator’s clinical judgment at the time of surgery, introducing a potential risk of selection bias. Furthermore, the retrospective nature of the study prevented a comprehensive evaluation of baseline comparability among the three groups. Crucial clinical variables—such as initial socket morphology, buccal plate thickness, specific reason for tooth extraction, baseline bone quality, exact implant dimensions (diameter and length), and specific healing intervals—could not be completely standardized or adequately compared. Without demonstrating strict baseline comparability, there is a substantial risk of confounding, making it difficult to definitively attribute the observed differences in marginal bone loss solely to the applied socket preservation protocols. As reported, the study groups also differ considerably in age, sex distribution, and implant location (maxilla vs. mandible). The pooling of data from both jaws without a specific anatomical mapping of the keratinized tissue width may introduce additional confounding variability, despite all sites clinically presenting with adequate soft tissue conditions at baseline. Most notably, the implant systems were unevenly distributed, with the Control Group receiving exclusively one implant type (Straumann BLT), while the test groups received a mix of two different systems. Although both implant lines share comparable macro-geometries, internal connections, and platform-switching configurations, these baseline discrepancies make it difficult to determine whether the observed differences in marginal bone loss are solely attributable to the socket preservation protocol or if they are influenced by these other variables. Furthermore, regarding the secondary outcome, insertion torque values from these two distinct implant systems were pooled for analysis; this approach might obscure minor system-specific differences in primary stability. The potential impact of these confounding factors on peri-implant bone remodeling requires that the present findings be interpreted with extreme caution. Therefore, while these initial 5-year results support the use of slow-resorbing membranes to improve crestal bone preservation, further prospective investigations with larger sample sizes and longer follow-up are required to confirm these findings.

## 5. Conclusions

Within the limitations of this retrospective pilot study, the preliminary observations provide a descriptive trend regarding the potential role of barrier membrane selection in 5-year peri-implant marginal bone stability. The application of a slow-resorbing bovine pericardium membrane, combined with a heterologous cortico-cancellous bone graft, recorded lower mean marginal bone loss values. Nevertheless, the extremely small sample size (*n* = 24) restricts the generalizability of these findings, and the absence of adequate statistical support precludes asserting the superiority of this protocol over rapidly degrading native collagen membranes or spontaneous healing. Given the strictly exploratory and hypothesis-generating nature of this pilot investigation, coupled with the very small sample size (8 implants per group) and the absence of inferential statistical analyses, these findings must be interpreted with extreme caution and do not support definitive clinical conclusions. Instead, they serve as a preliminary indicator to guide the direction of future research in this field. Further well-designed, prospective randomized clinical trials with larger patient cohorts and extended follow-up periods are necessary to thoroughly investigate these long-term outcomes and establish conclusive clinical guidelines for crestal bone maintenance under functional loading.

## Figures and Tables

**Table 1:** Summary of patient demographics and clinical site characteristics according to the treatment group. Age is reported as mean ± standard deviation (SD). Gender is reported as male (M) and female (F). Implant locations are categorized into maxillary and mandibular arches.

Group	N. Patients (Implants)	Age (Mean ± SD)	Gender	Position (Maxilla/Mandible)	Implant Type
Test Group 1	8	47.9 ± 8.5	3 M/5 F	5 Maxilla/3 Mandible	3 BTK IS+/5 Straumann BLT
Test Group 2	8	50.9 ± 9.4	3 M/5 F	5 Maxilla/3 Mandible	6 BTK IS+/2 Straumann BLT
Control Group	8	56.8 ± 11.6	7 M/1 F	3 Maxilla/5 Mandible	0 BTK IS+/8 Straumann BLT

**Table 2:** Summary of implant characteristics and clinical outcomes in Test Group 1, Test Group 2, and the Control Group, including insertion torque, marginal bone loss (MBL), implant survival, and complications. Data are reported as mean ± SD or *n* (%).

Variable	Test Group 1 (*n* = 8)	Test Group 2 (*n* = 8)	Control Group (*n* = 8)
Site ISQ Mean ± SD	68.38	66.25	65.50
Torque (Ncm) Mean ± SD	28.75	26.88	25.00
Mean MBL (mm) Mean ± SD	0.17 ± 0.20	0.46 ± 0.12	0.46 ± 0.27
Implant Survival	8/8 (100%)	8/8 (100%)	8/8 (100%)
Complications, *n* (%)	1 (12.5%)	1 (12.5%)	2 (25.0%)

**Table 3:** Summary of clinical and radiographic outcomes in the study groups.

Variable	Test Group 1	Test Group 2	Control Group
MBL Mesial (mm) Mean ± SD	0.12 ± 0.15	0.40 ± 0.18	0.41 ± 0.35
MBL Distal (mm) Mean ± SD	0.23 ± 0.25	0.53 ± 0.14	0.50 ± 0.32
Mean MBL (mm) Mean ± SD	0.17 ± 0.20	0.46 ± 0.12	0.46 ± 0.27

**Table 4:** Group-level descriptive statistics for marginal bone loss (MBL) at the 5-year follow-up. Values are presented as mean ± standard deviation (SD) and 95% confidence interval (CI).

Group	Treatment Protocol	Mean MBL (mm)	SD	95% CI
Test Group 1	Biomaterial + membrane (Pericardium)	0.17	0.20	0.00–0.34
Test Group 2	Biomaterial + membrane (Collagen)	0.46	0.12	0.36–0.57
Control Group	Spontaneous Healing	0.45	0.27	0.23–0.68

**Table 5:** Descriptive statistics of insertion torque values recorded at implant placement. Values are presented as mean ± standard deviation (SD).

Group	n	Mean Insertion Torque (Ncm)	SD
Test Group 1	8	28.75	6.94
Test Group 2	8	26.88	5.94
Control Group	8	25.00	6.55

## Data Availability

The original contributions presented in this study are included in the article. Further inquiries can be directed to the corresponding author.
